# Retraction: Kannan et al. AEG-1/miR-221 Axis Cooperatively Regulates the Progression of Hepatocellular Carcinoma by Targeting PTEN/PI3K/AKT Signaling Pathway. *Int. J. Mol. Sci.* 2019, *20*, 5526

**DOI:** 10.3390/ijms23126606

**Published:** 2022-06-14

**Authors:** International Journal of Molecular Sciences Editorial Office

**Affiliations:** MDPI, St. Alban-Anlage 66, 4052 Basel, Switzerland; ijms@mdpi.com

The journal retracts the article, “AEG-1/miR-221 Axis Cooperatively Regulates the Progression of Hepatocellular Carcinoma by Targeting PTEN/PI3K/AKT Signaling Pathway” [1], cited above.

We have been made aware that a number of figures in the paper cited above [1] contain manipulation. Several images from Figures 4 and 5 are unexpectedly similar. In accordance with our ethics procedures, an investigation was conducted. The authors have not been able to provide a satisfactory explanation for these irregularities, which brings uncertainty regarding the scientific conclusions. Therefore, to ensure the addition of only high-quality scientific works to the field of scholarly publication, this paper [1] has now been retracted and shall be marked accordingly. The authors agree to this retraction.

We apologize to our readership that this went undetected until now.

## References

[1] Kannan M., Jayamohan S., Moorthy R.K., Chabattula S.C., Ganeshan M., Arockiam A.J.V. (2019). AEG-1/miR-221 Axis Cooperatively Regulates the Progression of Hepatocellular Carcinoma by Targeting PTEN/PI3K/AKT Signaling Pathway. Int. J. Mol. Sci..

